# Renal Resistive Index as a Novel Predictor of Contrast-Induced Acute Kidney Injury in Cirrhosis

**DOI:** 10.7759/cureus.110491

**Published:** 2026-06-08

**Authors:** Jesse Jacob Skariah, Rushil Solanki, Jithin John, Akhil N V, Prasanth T S, Antony George, Jijo Varghese, Krishnadas Devadas

**Affiliations:** 1 Gastroenterology and Hepatology, Government Medical College, Thiruvananthapuram, IND; 2 Gastroenterology and Hepatology, Ansh Clinic, Ahmedabad, IND; 3 Gastroenterology, Government Medical College, Thiruvananthapuram, IND; 4 Gastroenterology and Hepatology, N.S. Cooperative Hospital, Kollam, IND

**Keywords:** cirrhosis, computed tomography (ct) scan, contrast-induced acute kidney injury (ci-aki), creatinine, imaging, iodinated contrast (ic), meld-na, renal resistive index (rri)

## Abstract

Background: Patients with liver cirrhosis undergo repeated contrast-enhanced imaging procedures. We investigated the risk factors for contrast-induced acute kidney injury (CI-AKI) in cirrhotic patients with normal renal function.

Methods: A prospective observational cohort study included 110 consecutive patients with cirrhosis who underwent iodinated contrast (IC)-based imaging procedures. Baseline demographic, clinical, and laboratory data and Renal Resistive Index (RRI) were recorded prior to imaging. All patients were assessed for the development of CI-AKI 48-72 hours after contrast administration.

Results: A total of 34 (30.9%) patients developed CI-AKI after contrast administration. Prior history of acute kidney injury (AKI) was seen in 18 (52.9%) patients who developed CI-AKI and 4 (5.3%) patients who did not develop CI-AKI (p < 0.001). Alcohol-related liver disease was the major etiological factor of cirrhosis in 18 (52.9%) patients with CI-AKI as opposed to 23 (30.3%) patients without CI-AKI (p = 0.047). Baseline estimated glomerular filtration rate (eGFR) and serum creatinine levels were not associated with an increased risk of CI-AKI. Higher values of baseline RRI were seen in patients who developed CI-AKI. On univariate analysis, gender, prior history of AKI, grade III ascites, hepatic encephalopathy, Child-Turcotte-Pugh (CTP) score, Model for End-Stage Liver Disease-Sodium (MELD-Na), and Renal Resistive Index (RRI) were found to be significant risk factors for predicting CI-AKI. On multivariate analysis, Renal Resistive Index (RRI), CTP score, and MELD-Na score were associated with an increased risk of CI-AKI. Preprocedural RRI >0.62, CTP score >10, and MELD-Na >24 had significant predictive value for the occurrence of CI-AKI.

Conclusion: Advanced liver cirrhosis was associated with an increased risk of CI-AKI. RRI is an easily available and simple radiologic technique to predict the occurrence of CI-AKI among patients with liver cirrhosis with normal renal function tests.

## Introduction

Renal dysfunction is associated with morbidity and mortality in patients with liver cirrhosis [1,2]. Patients with cirrhosis have a significantly higher risk of renal failure than the general population, and the condition is seen in as many as 20% of hospitalized patients with cirrhosis. There are many causes of renal failure in cirrhosis, including intravascular volume depletion, diuretic use, large-volume paracentesis, and altered renal hemodynamics [3].

Portal hypertension leads to splanchnic vasodilation predominantly via nitric oxide-related mechanisms, resulting in increased peripheral vascular resistance, reduced mean arterial pressure, and arterial underfilling [4]. As a compensatory measure for the reduced perfusion pressure, endogenous vasoconstrictor mechanisms get activated, causing renal tubular reabsorption of sodium and renal vasoconstriction. The consequent neuro-humoral activation and renal vasoconstriction progress until glomerular filtration decreases and overt renal failure develops [5].

Contrast-induced acute kidney injury (CI-AKI) due to iodinated contrast agents is a commonly encountered cause of renal dysfunction. Iodinated contrast (IC) toxicity is postulated to occur predominantly at the outer renal medulla, which is a region with limited blood supply and intense tubular transport activity. The passage of IC increases the metabolic activity in the renal medulla, hence causing increased circulatory demand. Subtle renal circulatory abnormalities associated with cirrhosis have the potential to overwhelm the auto-regulatory mechanisms and precipitate kidney dysfunction [6-8]. Chronic liver disease and cirrhosis are associated with an array of complications, and many of these, including portal vein thrombosis, hepatocellular carcinoma, and other malignancies, necessitate the use of iodinated contrast (IC) agents for diagnosis and treatment [9].

Although cirrhosis has been suggested as a risk factor for CI-AKI [9], only a few studies, to date, have specifically investigated this issue. A prospective study by Guevara et al. [10] did not find an increased susceptibility to CI-AKI in cirrhotic patients. Although this was a comprehensive study, it was conducted under idealized circumstances. Our goal was to conduct a study that may be more representative of the cirrhotic patients we encounter in the routine clinical settings. The study by Najjar et al. [9] was a retrospective study that also did not find an increased prevalence of CI-AKI in cirrhotic patients. A recent study by Ul Abideen et al. observed a frequency of 5.1% for CI-AKI in patients with cirrhosis; however, the authors did not precisely define CI-AKI, making it difficult to interpret the results of the study [11]. We aimed to assess the prevalence of CI-AKI in cirrhotic patients and identify the predictors of CI-AKI in liver cirrhosis. We thus conducted this study among cirrhotic patients who received IC as a part of an imaging procedure for further analysis to identify characteristics and specific risk variables. This may be useful for prior precautions and early management in high-risk patients for this condition. This article was presented as a scientific poster at the annual conference of the Indian Society of Gastroenterology (ISG-CON), held in Bangalore, India, in December 2023, and was published as a conference poster abstract in a supplementary issue of the society’s journal.

## Materials and methods

Study design

This single-center prospective observational cohort study was performed at a tertiary care teaching hospital in South India over six months from May 2022 to November 2022.

Study participants

All consecutive patients with cirrhosis who underwent intravenous iodinated contrast imaging were included. Liver cirrhosis was diagnosed by standard imaging and biochemical criteria. Demographic, clinical, and laboratory variables were obtained at baseline. Patients with pre-existing acute kidney injury or chronic kidney disease (CKD) were excluded from the study. Similarly, patients without a prior established diagnosis of CKD but a baseline serum creatinine value more than 1.5 mg/dL were excluded from the study.

Ethical considerations

Human Ethics Committee of Medical College, Thiruvananthapuram, India, clearance (HEC NO: 04/18/2022/MCT, dated: 31-05-2022) was obtained for the study.

Data collection and variables

All patients in the study received the same standard amount of IV contrast (1.2 mL/kg of Iohexol). The serum creatinine and renal function test values were noted before and 48-72 hours post-contrast administration to assess for contrast-induced acute kidney injury. The estimated glomerular filtration rate (eGFR) was calculated based on Modification of Diet in Renal Disease study-6 (MDRD-6) equation [12,13]. Model for End-Stage Liver Disease-Sodium (MELD-Na) scores [14,15] and Child-Turcotte-Pugh (CTP) grades [16] of cirrhosis were estimated for all patients. Contrast-induced acute kidney injury (CI-AKI ) was defined by the Kidney Disease Improving Global Outcomes (KDIGO) consensus definition [17].

Renal vasoconstriction was assessed using Doppler ultrasound of the renal arteries by using an index called Renal Resistive Index (RRI) [18]. Patients were made to lie in the supine position, right lateral, and left lateral position. Arcuate arteries (at the corticomedullary junction) and interlobar arteries (adjacent to medullary pyramids) were identified. Doppler evaluation of the renal arteries was done using a 3.5 MHz convex transducer by two independent examiners. Renal Resistive Index (RRI) was determined using the formula: RRI = (Peak systolic flow- Peak diastolic flow)/Peak systolic flow.

A minimum of five reproducible waveforms from each kidney were obtained, and RRIs from these waveforms were averaged to arrive at mean RRI values for each kidney. All these recordings were made by a single operator, and multiple readings (minimum of five) were taken which helps to remove inter-observer variability. RRI was obtained prior to contrast administration [19].

Statistical analysis

Data entry was done using Microsoft Excel (Microsoft Corporation, Redmond, Washington, United States) and then imported to IBM SPSS version 27 (IBM Corp., Armonk, New York, United States) for analysis. Quantitative data were expressed using mean (with standard deviation) and median (with interquartile range), and categorical data were expressed as proportions and percentages. Comparisons of continuous variables between study groups were made using the Student t-test for normally distributed data, the Mann-Whitney test for non-parametric data, and the chi-squared test for analysis of categorical variables. A multivariate analysis including variables with significant predictive value in the univariate analysis was performed using binary logistic regression to determine the variables with independent predictive value. The best cut-offs of prediction were obtained from receiver operator characteristic (ROC) curves and Youden's Index analysis.

## Results

Baseline demographic and clinical data

A total of 110 patients were included in the final analysis. The mean age of the study population was 57.95 ± 9.8 years. Seventy-two patients (65.5%) were males, and 38 (34.5%) were females. Twenty-two patients (20.2%) had a history of prior AKI. Twenty-eight patients (25.4%), 40 patients (36.4%), and 42 patients (38.2%) belonged to the Child A, Child B, and Child C categories, respectively.

On etiological workup for cirrhosis, 49 (44.5%) had non-alcoholic fatty liver disease/steatohepatitis (NAFLD/NASH), 41 (37.3%) had alcohol-related liver disease, and 13 (11.8%) had chronic viral hepatitis. Fifty-seven patients (51.8%) had type 2 diabetes mellitus. Of the patients studied, 99 (90%) were on beta blockers and 41 (37.3%) were on diuretics.

Identifying factors associated with increased risk of CI-AKI

Thirty-four patients (30.90%) developed CI-AKI. Twenty-seven males (37.5%) developed CI-AKI, a significantly higher proportion than females (18.4%) (p = 0.039). Eighteen patients (52.9%) with a prior history of AKI developed CI-AKI compared to four patients (5.3%) without prior AKI (p = 0.001). On univariate analysis, age of the patient, diabetes status, systemic hypertension, hypothyroidism, coronary artery disease, cerebrovascular events, chronic obstructive pulmonary disease, obesity, and frailty were not associated with increased incidence of CI-AKI.

Among patients who developed CI-AKI, seven patients (20.6%) had international normalized ratio (INR) above 2.3, and nine patients (26.5%) had INR between 1.7 and 2.3, while the remaining patients had INR <1.7. Among those who did not have CI-AKI, 67 (88.7%) had INR <1.7, and no patient had an INR >2.3 (p < 0.001). In patients with CI-AKI, eight patients (23.5%) had baseline urea levels > 40 mg/dL as opposed to only six patients (7.9%) who did not develop CI-AKI (p = 0.009). Grade III ascites was present in 13 patients (38.2%) with CI-AKI, compared with eight patients (10.5%) without CI-AKI (p=0.006). Alcoholic liver disease was the etiological factor in 18 (52.9%) and 41 (30.3%) patients with and without CI-AKI, respectively (p = 0.047).

Twenty-one patients (61.8%) who developed CI-AKI had Child C Cirrhosis compared to 21 patients (27.6%) in the non-CI-AKI group (p = 0.003). Hepatic encephalopathy was seen in 12 patients (35.4%) with CI-AKI as opposed to nine patients (11.8%) in the non-CI-AKI group (p = 0.004). There was no statistically significant difference among the rates of GI bleed, prior spontaneous bacterial peritonitis, urinary tract infection, cellulitis, lower respiratory tract infection, hepatocellular carcinoma, and portal vein thrombosis between the two groups. Interestingly, being on diuretics or beta-blocker therapy did not seem to be associated with a higher risk of CI-AKI. Also, the urine protein-creatinine (PC) ratio was not significantly elevated among patients who developed CI-AKI.

Higher values of baseline RRI were seen in patients who developed CI-AKI. Twenty-five patients (73.5%) with CI-AKI had an RRI >0.65 compared to six patients (7.9%) without CI-AKI (p < 0.001). On univariate analysis, gender, prior history of AKI, Grade III ascites, hepatic encephalopathy, Child-Turcotte-Pugh (CTP) score, Model for End-Stage Liver Disease-Sodium (MELD-Na) and Renal Resistive Index (RRI) were found to be significant risk factors for predicting CI-AKI. But on multivariate analysis, the significant predictors of CI-AKI were CTP score (p = 0.0008), MELD-Na (p = 0.0001), and RRI (p < 0.0001) (Table 1).

**Table 1:** Binary logistic regression mode (Final) for CI-AKI MELD-Na: Model for End-Stage Liver Disease-Sodium, RRI: Renal Resistive Index, B: beta coefficient, S.E.: standard error, Wald: Wald statistic, df: degrees of freedom, p: p value, OR: odds ratio, CI: confidence interval.

	B	S.E.	Wald	df	p	OR	95% CI for OR
MELD-Na	2.655	0.884	9.012	1	0.003	14.2	2.5-80.5
CHILD-C	1.524	0.798	3.646	1	0.056	4.6	1-22
RRI	2.835	0.817	12.053	1	0.001	17	3.4-84.4

**Figure 1:**
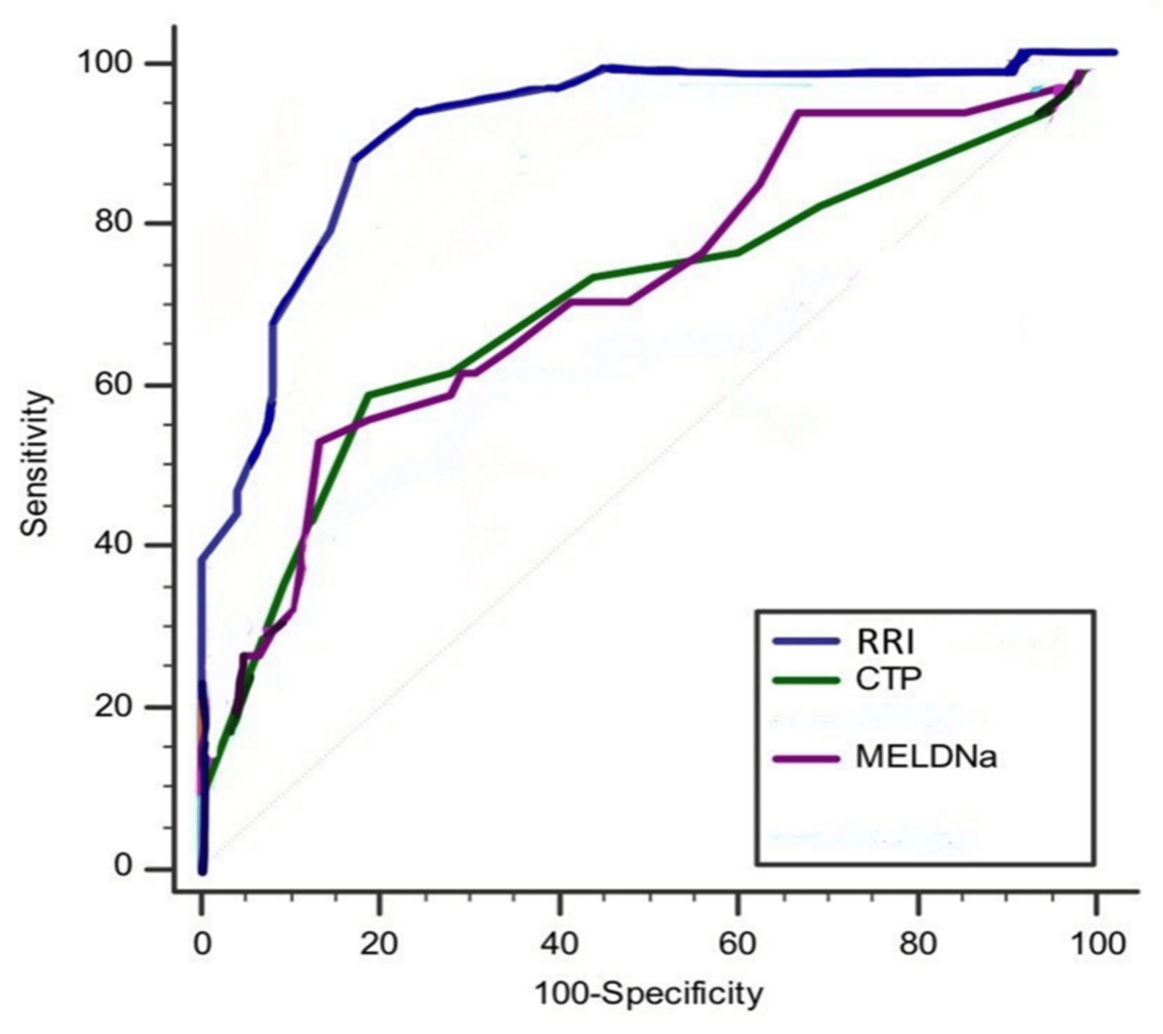
Area under the curve (AUC) plot of predictive factors for CI-AKI CI-AKI: contrast-induced acute kidney injury, RRI: Renal Resistive Index, CTP: Child-Turcotte-Pugh, MELD-Na: Model for End-Stage Liver Disease-Sodium.

To predict CI-AKI, optimum cut-off value of RRI was >0.62 (area under receiver operator curve (AUROC): 0.921; 95% CI: 0.854-0.964), MELD-Na was >24 (AUROC: 0.721; 95% CI: 0.628-0.802), and CTP score was >10 (AUROC: 0.699; 95% CI: 0.604-0.783) (Table 2). An RRI cut-off of >0.62 demonstrated a sensitivity of 88.24% and a specificity of 86.84%, outperforming traditional severity scores of liver disease including MELD-Na (area under the curve (AUC): 0.721) and CTP (AUC: 0.699) (Figure 1).

**Table 2:** Predictive factors for CI-AKI CI-AKI: contrast-induced acute kidney injury, CTP: Child-Turcotte-Pugh score, MELD-Na: Model for End-Stage Liver Disease-Sodium score, RRI: Renal Resistive Index, ROC: receiver operator characteristic, LR: likelihood ratio, -PV: negative predictive value, +PV: positive predictive value.

Variable	CTP	MELD-Na	RRI
Classification variable	CI-AKI	CI-AKI	CI-AKI
Total	110		
CI-AKI: Yes	34		
CI-AKI: No	76		
Area under the ROC curve (AUC)	0.699	0.721	0.921
Standard error	0.0595	0.0552	0.0302
95% CI	0.604-0.783	0.628-0.802	0.854-0.964
z statistic	3.345	4.005	13.925
p	0.0008	0.0001	<0.0001
Youden index J	0.404	0.3978	0.7508
Optimum cut-off	>10	>24	>0.62
Sensitivity	58.82	52.94	88.24
Specificity	81.58	86.84	86.84
+LR	3.19	4.02	6.71
-LR	0.5	0.54	0.14
+PV	58.8	64.3	75
-PV	81.6	80.5	94.3

## Discussion

Contrast-induced renal dysfunction has not been studied extensively in patients with liver cirrhosis. Based on currently available data, it is difficult to ascertain whether the presence and severity of cirrhosis are predisposing factors for CI-AKI. Hemodynamic alterations and bacterial translocation, which occur in cirrhosis of the liver, may play a role in the development of CI-AKI.

The frequency of CI-AKI in patients undergoing iodinated contrast-based imaging procedures in our study was 30.90%. In previous studies done by Ul Abideen et al. [11] and Safi et al. [20], the incidence of CI-AKI was 17.9% and 5.1%, respectively. Such varying estimates could be due to the slightly different definitions of CI-AKI used and variations in the inclusion criteria employed. An earlier study concluded a 25% frequency of CI-AKI in patients with cirrhosis [21].

There was no significant association between CI-AKI and age, similar to findings reported in most previous studies [11,20,21]. Although in univariate analysis males had a significantly higher incidence of CI-AKI compared to females, multivariate analysis did not find any significant difference. The incidence of CI-AKI was not higher in patients on diuretics or beta blockers. This finding is in agreement with a previous study done by Lodhia et al. on hospitalized patients with cirrhosis [22]. The CTP and MELD-Na scores are widely used for the assessment of prognosis in liver cirrhosis. Patients who developed CI-AKI generally had higher CTP and MELD-Na scores than those without CI-AKI. This would imply that the former had an overall worse liver functional status. In advanced stages of cirrhosis, there is fixed resistance from hepatic fibrosis and dynamic resistance in the splanchnic arteries due to a) vasodilators such as nitric oxide, carbon monoxide, and endogenous cannabinoids; and b) vasodilation due to inflammatory cytokines (tumor necrosis factor-alpha, interleukin-6) induced by bacterial translocation from the gut.

Splanchnic vasodilatation leads to pooling of blood in the splanchnic circulation and decreased effective blood volume. In this situation, the compensatory increase in cardiac output via activation of the sympathetic nervous system by carotid baroreceptors maintains renal perfusion. However, the compensatory increase in cardiac output is insufficient to maintain appropriate renal perfusion and circulatory blood volume when cirrhosis decompensates, and portal hypertension worsens. This issue is exacerbated by the fact that up to 40%-50% of people with cirrhosis develop cirrhotic cardiomyopathy. The renin-angiotensin-aldosterone system is activated by decreased renal perfusion as liver disease severity increases, leading to extra-splanchnic vasoconstriction and water and salt retention.

More advanced stages of cirrhosis are associated with the difference in mean values of bilirubin and albumin, which were not statistically significant in both groups; however, INR values were significantly higher in the CI-AKI group (p < 0.001), with over 20% of patients with CI-AKI having an INR > 2.3. This association with INR has been reported in various previous studies [21]. The frequency of Grade III ascites and hepatic encephalopathy was significantly higher in patients with CI-AKI, and these factors may contribute to the higher CTP scores in these patients. Higher INR, CTP score, and MELD-Na scores indicate poor liver functional status, which may be associated with systemic vasodilatation and compromised renal perfusion, hence predisposing to CI-AKI. Also, Grade III ascites and hepatic encephalopathy, which indicate worse liver functional status, were associated with a higher risk of CI-AKI in univariate analysis. Similar findings were observed in previous studies by Ul Abideen et al. [11] and Safi et al. [20]. The higher these scores, the worse the prognosis and mortality in advanced liver disease. An important finding of our study was that patients who developed CI-AKI had worse CTP and MELD-Na scores as compared to those who did not have CI-AKI. This generally means that the former had more severe liver dysfunction and a worse prognosis. This finding is further supported by the fact that the mean INR was higher in the CI-AKI group. A higher INR indicates a poorer synthetic function of the liver. These results may have important implications. Patients with severe cirrhosis and worse prognostic scores may be more susceptible to CI-AKI; this again may be due to a combination of an abnormal circulatory and hormonal milieu, as described earlier.

An interesting observation in our study was that baseline values of creatinine or eGFR were not associated with the development of CI-AKI. Since patients with established CKD were excluded from the study, the study population had a baseline creatinine within normal limits (serum creatinine: ≤1.5 mg/dL), hence implying that a higher baseline creatinine up to 1.5 mg/dL did not increase the risk of CI-AKI. Similar findings were obtained from a study on patients with active cancer undergoing contrast-enhanced computed tomography studies in South Korea [23]. Another observation was that those who had baseline urea levels > 40 mg/dL had an increased risk of CI-AKI. Higher baseline urea levels may indicate a subclinical intravascular volume contracted state, which may predispose to CI-AKI.

The cut-off value of RRI for predicting CI-AKI in our study was >0.62. In a study by Xu et al. in patients undergoing percutaneous coronary intervention (PCI), the cut-off value for CI-AKI was >0.69 [24]. The patients who developed CI-AKI in our study have high-normal RRI values. This may be due to generalized systemic vasodilatation in advanced liver cirrhosis. High RRI values indicate increased renal vascular resistance, and it has been assessed as a marker of endothelial vascular damage in cardiovascular diseases [25]. Normal RRI values in adults range from 0.47 to 0.70 [19]. Changes in intrarenal arterial waveforms were shown to be associated with urinary obstruction, several types of intrinsic renal disorders, and renal vascular disease [19]. The highest values of RRI in previous studies on patients with renal disease were seen in those with vascular or interstitial disease patterns as opposed to glomerular pathology [19]. The ability of Doppler sonography to identify latent hepatorenal syndrome before liver transplantation was shown by the University of Michigan group [26].

Various forms of kidney injury in cirrhosis, including hepatorenal syndrome, have similar pathophysiological mechanisms, and the utility of RRI has been demonstrated in these pathologies in previous studies [26]. The increased RRI value in our study was mainly influenced by the tendency toward lower intrarenal end-diastolic volume among patients who developed CI-AKI. It is possible that greater RRI values reflect increased intrarenal vascular resistance related to endothelial dysfunction [27] or possibly even renal arteriosclerosis. This baseline augmented intrarenal vascular resistance may act permissively to the tubular injury caused by highly concentrated viscous iodinated contrast media in renal outer medulla [27]. Iodinated contrast agents may cause an imbalance between vasodilative and vasoconstrictive agents [28]. The vasoconstriction of afferent arterioles mediated by adenosine and triggered by contrast-induced stimulation of tubule-glomerular feedback may also contribute to increased renal vascular resistance [29]. All these findings are in agreement with the observed propensity toward increased RRI in subjects exhibiting CI-AKI. In a previous study, invasive measurement of intrarenal blood flow directly before and after iodinated contrast administration revealed a significant increase in resistance index (defined as the ratio of mean blood pressure and average peak renal velocity) and a decrease in peak systolic velocity [29].

To our knowledge, this represents the first study that demonstrates the use of RRI as a predictive tool to assess the risk of CI-AKI in cirrhotic patients specifically. In multivariate analysis, CTP score, MELD-Na, and RRI were independent predictors for CI-AKI. The predictive value of pre-procedure RRI for CI-AKI has been observed in other studies in patients undergoing percutaneous coronary intervention (PCI) for coronary artery disease, and this study provides objective evidence to extend this sound technical principle to the monitoring of CI-AKI risk in patients with cirrhosis. The study was limited by the fact that it was conducted on subjects from a single center with a limited sample size. Also, an extended follow-up of patients who developed CI-AKI was not included in this study.

## Conclusions

There is a high incidence of CI-AKI in patients with cirrhosis as compared to the general population. Advanced stages of cirrhosis may be associated with increased risk for CI-AKI. Pre-contrast RRI, MELD-Na, and CTP scores may be useful to predict the occurrence of CI-AKI in patients with cirrhosis and apparently normal renal function as determined by serum creatinine values. RRI holds promise as a novel non-invasive bedside tool for monitoring the risk of CI-AKI in patients with cirrhosis and may aid in initiating closer monitoring and preventive strategies for the same. Patients who have higher values of RRI and those with advanced liver disease may benefit from careful volume expansion and hydration strategies as per protocols to prevent contrast-related kidney injury.

## References

[1] Sobhani I, Lehy T, Laurent-Puig P, Cadiot G, Ruszniewski P, Mignon M (1993). Chronic endogenous hypergastrinemia in humans: evidence for a mitogenic effect on the colonic mucosa. Gastroenterology.

[2] Wu CC, Yeung LK, Tsai WS (2006). Incidence and factors predictive of acute renal failure in patients with advanced liver cirrhosis. Clin Nephrol.

[3] du Cheyron D, Bouchet B, Parienti JJ, Ramakers M, Charbonneau P (2005). The attributable mortality of acute renal failure in critically ill patients with liver cirrhosis. Intensive Care Med.

[4] Epstien M (1977). Deranged renal function in liver disease. Contr Nephrol.

[5] Moreau R, Lebrec D (2003). Acute renal failure in patients with cirrhosis: perspectives in the age of MELD. Hepatology.

[6] From AM, Bartholmai BJ, Williams AW, Cha SS, McDonald FS (2008). Mortality associated with nephropathy after radiographic contrast exposure. Mayo Clin Proc.

[7] Mathew R, Haque K, Woothipoom W (2006). Acute renal failure induced by contrast medium: steps towards prevention. BMJ.

[8] Barrett BJ, Parfrey PS (1994). Prevention of nephrotoxicity induced by radiocontrast agents. N Engl J Med.

[9] Najjar M, Hamad A, Salameh M, Agarwal A, Feinfeld DA (2002). The risk of radiocontrast nephropathy in patients with cirrhosis. Ren Fail.

[10] Guevara M, Fernández-Esparrach G, Alessandria C (2004). Effects of contrast media on renal function in patients with cirrhosis: a prospective study. Hepatology.

[11] Ul Abideen Z, Mahmud SN, Salih M, Arif A, Ali F, Rasheed A, Zafran M (2018). Contrast-induced acute kidney injury in patients with liver cirrhosis: a retrospective analysis. Cureus.

[12] Levey AS, Coresh J, Greene T (2006). Using standardized serum creatinine values in the modification of diet in renal disease study equation for estimating glomerular filtration rate. Ann Intern Med.

[13] Levey AS, Bosch JP, Lewis JB, Greene T, Rogers N, Roth D (1999). A more accurate method to estimate glomerular filtration rate from serum creatinine: a new prediction equation. Ann Intern Med.

[14] Kim WR, Biggins SW, Kremers WK (2008). Hyponatremia and mortality among patients on the liver-transplant waiting list. N Engl J Med.

[15] Alessandria C, Ozdogan O, Guevara M (2005). MELD score and clinical type predict prognosis in hepatorenal syndrome: relevance to liver transplantation. Hepatology.

[16] Pugh RN, Murray-Lyon IM, Dawson JL, Pietroni MC, Williams R (1973). Transection of the oesophagus for bleeding oesophageal varices. Br J Surg.

[17] (2012). Notice. Kidney Int Suppl.

[18] Pourcelot L (1974). Clinical applications of transcutaneous Doppler examination. Doppler Ultrasound Velocimetry.

[19] Tublin ME, Bude RO, Platt JF (2012). The resistive index in renal Doppler sonography: where do we stand?. AJR Am J Roentgenol.

[20] Safi W, Rauscher I, Umgelter A (2015). Contrast-induced acute kidney injury in cirrhotic patients: a retrospective analysis. Ann Hepatol.

[21] Wybraniec MT, Bożentowicz-Wikarek M, Chudek J, Mizia-Stec K (2017). Pre-procedural renal resistive index accurately predicts contrast-induced acute kidney injury in patients with preserved renal function submitted to coronary angiography. Int J Cardiovasc Imaging.

[22] Lodhia N, Kader M, Mayes T, Mantry P, Maliakkal B (2009). Risk of contrast-induced nephropathy in hospitalized patients with cirrhosis. World J Gastroenterol.

[23] Hong SI, Ahn S, Lee YS, Kim WY, Lim KS, Lee JH, Lee JL (2016). Contrast-induced nephropathy in patients with active cancer undergoing contrast-enhanced computed tomography. Support Care Cancer.

[24] Xu ZR, Chen J, Liu YH, Liu Y, Tan N (2019). The predictive value of the renal resistive index for contrast-induced nephropathy in patients with acute coronary syndrome. BMC Cardiovasc Disord.

[25] Lubas A, Kade G, Niemczyk S (2014). Renal resistive index as a marker of vascular damage in cardiovascular diseases. Int Urol Nephrol.

[26] Platt JF, Marn CS, Baliga PK, Ellis JH, Rubin JM, Merion RM (1992). Renal dysfunction in hepatic disease: early identification with renal duplex Doppler US in patients who undergo liver transplantation. Radiology.

[27] Filomia R, Maimone S, Caccamo G (2016). Acute kidney injury in cirrhotic patients undergoing contrast-enhanced computed tomography. Medicine.

[28] Liu ZZ, Schmerbach K, Lu Y (2014). Iodinated contrast media cause direct tubular cell damage, leading to oxidative stress, low nitric oxide, and impairment of tubuloglomerular feedback. Am J Physiol Renal Physiol.

[29] Kurihara O, Takano M, Uchiyama S (2015). Microvascular resistance in response to iodinated contrast media in normal and functionally impaired kidneys. Clin Exp Pharmacol Physiol.

